# A Service-Learning Project Based on a Community-Oriented Intelligent Health Promotion System for Postgraduate Nursing Students: Mixed Methods Study

**DOI:** 10.2196/52279

**Published:** 2023-12-15

**Authors:** Ting Sun, Xuejie Xu, Ningning Zhu, Jing Zhang, Zuchang Ma, Hui Xie

**Affiliations:** 1 School of Nursing Bengbu Medical College Bengbu, Anhui China; 2 Institute of Intelligent Machines Hefei Institutes of Physical Sciences Chinese Academy of Sciences Hefei, Anhui China

**Keywords:** service learning, intelligent health promotion system, scientific awareness, research innovation ability

## Abstract

**Background:**

Service learning (SL) is a pedagogical approach that combines community service with cognitive learning for professionals. Its efficacy in promoting community health has gained broad recognition in nursing education. The application of postgraduate nursing SL programs in community-based intelligent health remains underexplored. Thus, additional investigation is necessary to assess the influence of the SL project based on a community-oriented intelligent health promotion system (SLP-COIHPS) on postgraduate nursing students and health service recipients.

**Objective:**

This study aims to assess how SLP-COIHPS influences the scientific awareness and research innovation abilities of postgraduate nursing students. In addition, the study sought to examine the experiences of both participating students and health service recipients.

**Methods:**

We conducted a mixed methods investigation by using web-based surveys and conducting interviews. The web-based surveys aimed to explore the differences in scientific awareness and research innovation capabilities between 2 distinct groups: an experimental group of 23 postgraduate nursing students actively participated in SLP-COIHPS, while 23 postgraduate students (matched one-to-one with the experimental group in terms of grade, sex, and research methods) served as control participants. Semistructured interviews were conducted with 65% (15/23) of postgraduate students and 3% (12/405) of community residents who received health services, aiming to assess the project’s impact on them. The community-based intelligent health promotion system installed in intelligent health cabins can be conceptualized as an expert system providing valuable references for student health education. It has the capability to generate comprehensive assessments and personalized health guidance plans. Following training, students were involved in offering health assessments, health education, and related services. Subsequently, after the web-based surveys and semistructured interviews, quantitative data were analyzed using the SPSS (IBM Corp) software package, using 2-tailed *t* tests and Mann-Whitney *U* tests; qualitative data underwent analysis using the constructivist grounded theory approach.

**Results:**

Postgraduate nursing students participating in this program scored 12.83 (Cohen *d*>0.8; *P<*.001) and 10.56 (Cohen *d*>0.8; *P*=.004) points higher than postgraduate students in the control group in research awareness and research innovation capability, respectively. On the basis of the qualitative results, postgraduate students reported improvement in this program. Analysis of the interviews revealed a total of 12 subcategories across three primary domains: (1) specialized skills, (2) scientific research ability, and (3) comprehensive qualities. Community residents reported high satisfaction and positive experiences. Analysis of the interviews with community residents identified two primary categories: (1) satisfaction and (2) perceived benefits.

**Conclusions:**

SLP-COIHPS had a positive impact on students’ development of scientific awareness and research innovation ability. Qualitative study findings also support the further development of practical programs that integrate intelligent health and SL theories in the field of medical education. This includes exploring the potential factors influencing postgraduate nursing students’ research capabilities or investigating the long-term effects of the project.

## Introduction

### Background

Service learning (SL) originated from service volunteer programs in the United States in the 1960s. Robert Sigmon and William Ramsey further defined the concept in 1969 as an approach that balances the dual goals of meeting genuine human needs and fostering educational growth [1,2]. Today, SL is widely described as an approach that integrates community service with curricular learning through school-community partnerships. This allows students to engage in organized service initiatives to address community needs, develop problem-solving and critical analysis skills, and cultivate a sense of social responsibility in collaboration with peers and community members [3]. SL can be implemented in various forms, including investigation, planning, action, reflection, and demonstration or celebration [4].

SL has been widely adopted in the health sciences and nursing disciplines since its introduction in nursing education in the mid-1990s. It guides nursing and medical students to engage in health promotion practices across multiple settings and populations [5]. SL is a process that incorporates theory into the professional curriculum, with reflection as a necessary element. It focuses on the development of community activities, where professional teachers host service events for students in response to real-world community needs [6].

### Significance

Multiple studies have shown that SL experiences involving individuals, groups, and organizations can improve medical students’ academic and nonacademic performance, ethical and decision-making capabilities, and clinical self-efficacy [7-9]. Furthermore, SL significantly enhances medical students’ empathy, sense of social responsibility, and willingness to contribute to the community in their future medical practice [10-12]. Moreover, SL fosters collaboration and interprofessional teamwork, enabling future health care professionals to work more efficiently in different health care settings [13,14]. Hence, SL is an effective pedagogical approach that can help develop competent, responsible, and well-rounded health care professionals. SL has the potential to create mutually beneficial partnerships between schools and communities. However, there is a lack of studies investigating the impacts of SL from the viewpoint of the community or the service recipients [15].

Communities often serve as sites for SL. However, the integration of intelligent health promotion systems within communities, along with SL, is comparatively infrequent when compared with conventional community practices [16,17]. In recent years, intelligent health promotion systems have gained significant attention for providing personalized health advice and interventions to community residents using advanced technologies such as artificial intelligence, data analytics, and wearable devices. These systems have been shown to improve health outcomes and reduce chronic diseases [18-20]. Intelligent health promotion systems not only offer low-cost, safe, and reliable personalized health promotion services but also facilitate the exchange of critical information between numerous Internet of Things terminals and various health care service systems, thereby improving the efficiency and cost-effectiveness of health care services. Several studies have demonstrated the potential of intelligent health promotion systems in promoting population health and supporting health care [21-24]. For instance, Hu and Hao [23] found that a daily physical functioning and health promotion system based on nanoprotective technology effectively enhances the health of older adults and provides protection during exercise. Similarly, Sun et al [19] applied an intelligent, personalized, exercise prescription based on an eHealth promotion system to a community group of middle-aged and older people, resulting in significant improvements in some of the health outcomes of middle-aged and older community residents.

The importance of applying computer technology, mobile communication technology, Internet of Things, artificial intelligence, and big data in health promotion is gradually becoming evident. However, there is limited reporting about the use of SL in these fields. The most common approach is to have students collaborate in a web-based community setting, providing health care services [25-28]. Alternatively, researchers integrate the theory of SL into the development of a digital service product [29]. Until now, we can consider the SL project integrating the community smart health system as a form of e-SL. Enslein et al [30] defined e-SL as “a form of experiential education in electronic format, including electronically supported service-learning; it involves organized, focused experiential service-learning activities provided web-based, using the internet and state-of-the-art technology, allowing students, teachers, and community partners to collaborate remotely, promoting an increase in civic responsibility and meeting community needs.” e-SL combines SL with internet teaching (for this project, the system serves as a web-based coach), introducing new dynamics to electronic learning while applying knowledge to the real world [31]. For students, e-SL is akin to gaining on-site experience in a web-based setting, enhancing skills in applying knowledge, critical decision-making, leadership, time management, emotional intelligence, empathy, confidence, and social responsibility [32-35]. For community partners, additional personnel enable them to take on new projects, thereby expanding their service scope.

### Objectives

This study aimed to assess the influence of the service-learning project based on a community-oriented intelligent health promotion system (SLP-COIHPS) on the scientific awareness and research innovation capabilities of postgraduate nursing students. It also investigated the improvement of students’ professional competence or other skills and assessed the community residents’ evaluations and feedback regarding the services provided. This study will enhance the integration and improvement of intelligent health promotion systems and postgraduate SL. In addition, it will further the innovative development of postgraduate nursing education and practice, offering a valuable reference.

## Methods

### Study Aim and Design

To better understand the extent to which the project influences the research capabilities of postgraduate nursing students, explore other potential impacts, and comprehensively investigate the project’s effectiveness from the perspective of both postgraduate nursing students and service recipients, this study used a mixed methods design. Quantitative research methods were used to assess the impact of the program on the scientific awareness and research innovation capabilities of postgraduate nursing students. In addition, qualitative research methods, specifically semistructured interviews, were used to investigate the experiences of postgraduate nursing students and health service recipients who participated in the program.

### Instruments

In this study, to investigate the impact of this training model on the scientific awareness and research innovation capabilities of postgraduate nursing students, we selected questionnaires suitable for the study participants, which have previously been validated in the postgraduate student population.

#### Scientific Awareness Questionnaire for Postgraduate Students

We used a survey questionnaire developed by Zhang [24] consisting of 18 items, with a total score of 90. It covers 6 dimensions: problem identification awareness, problem value argumentation awareness, problem proposal awareness, doubt awareness, problem exploration awareness, and innovation awareness. Each dimension contained 3 questions, all scored using a 5-point Likert scale (1-5). The scores of the surveyed individuals were positively correlated with their research awareness levels. The questionnaire demonstrated a Cronbach α coefficient of .865, indicating good internal consistency.

#### Research Innovation Capabilities Questionnaire for Postgraduate Students

We used the Innovative Research Abilities Questionnaire developed by Zhang and Yang [36], comprising 4 dimensions: ideational innovation, methodological innovation, applicative innovation, and research innovation achievements. A 5-point Likert scale was used for negative scoring (5-1) in the first 3 dimensions, with a total of 11 items available. The research innovation achievements section encompassed 23 items. The overall score ranged from 0 to 100, with high scores indicative of great research innovation proficiency. The original questionnaire possessed a Cronbach α coefficient of .880, whereas in this study, the Cronbach α coefficient stands at .819.

### Development of the SL Project Based On a Community-Oriented Intelligent Health Promotion System

#### Overview

This study developed an SLP-COIHPS. In contrast to traditional community SL projects and web-based, community-based SL projects, this project seamlessly transitioned between real community health service scenarios and web-based community scenarios (such as WeChat public accounts or other social media platforms). It leveraged various digital technologies to provide web-based and offline services to patients and involved postgraduate nursing students in the entire process of developing, implementing, and enhancing digital health service products. This immersion allowed them to discover research challenges during the product development or health care service process, enhance their research capabilities, and improve their professional competence and overall qualities.

#### Community-Oriented Intelligent Health Promotion System

In 2018, the School of Nursing at Bengbu Medical College, in collaboration with the Hefei Institutes of Physical Sciences, Chinese Academy of Sciences, and 3 community health service centers, signed a tripartite agreement to collectively establish this project. The system was collaboratively developed by the teachers and students from these universities and research institutes engaged in interdisciplinary research at the convergence of medicine and engineering. It is subject to continuous iterative improvements and functional expansions.

**Figure 1:**
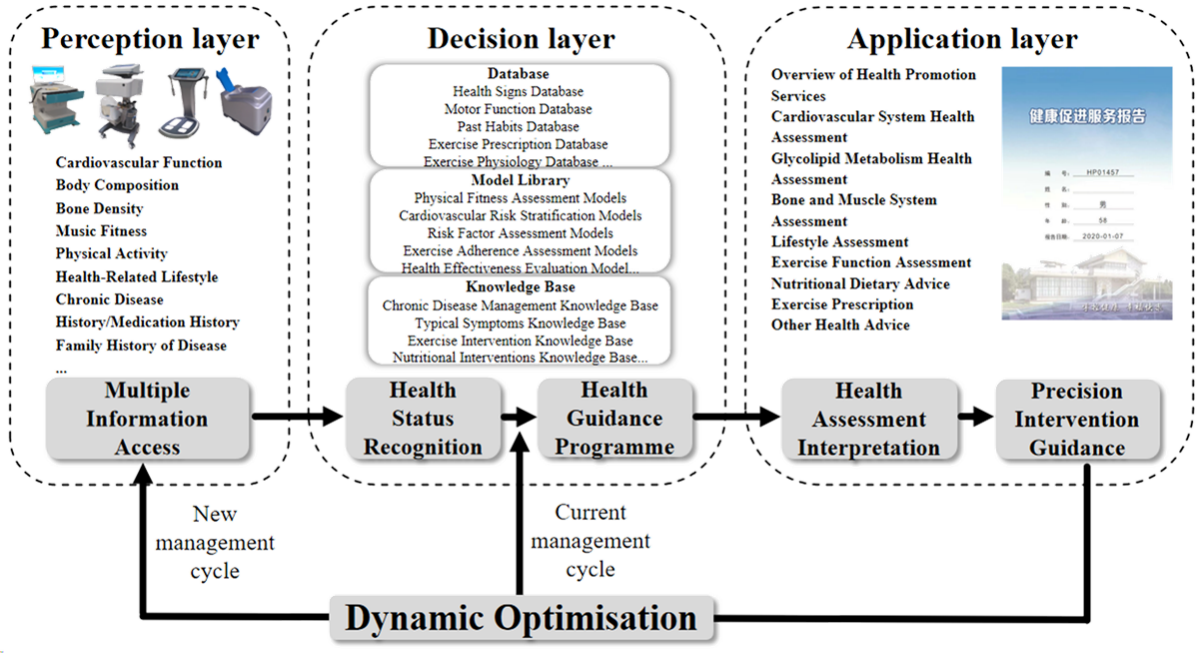
Diagram of the intelligent health promotion system’s architecture.

**Figure 2:**
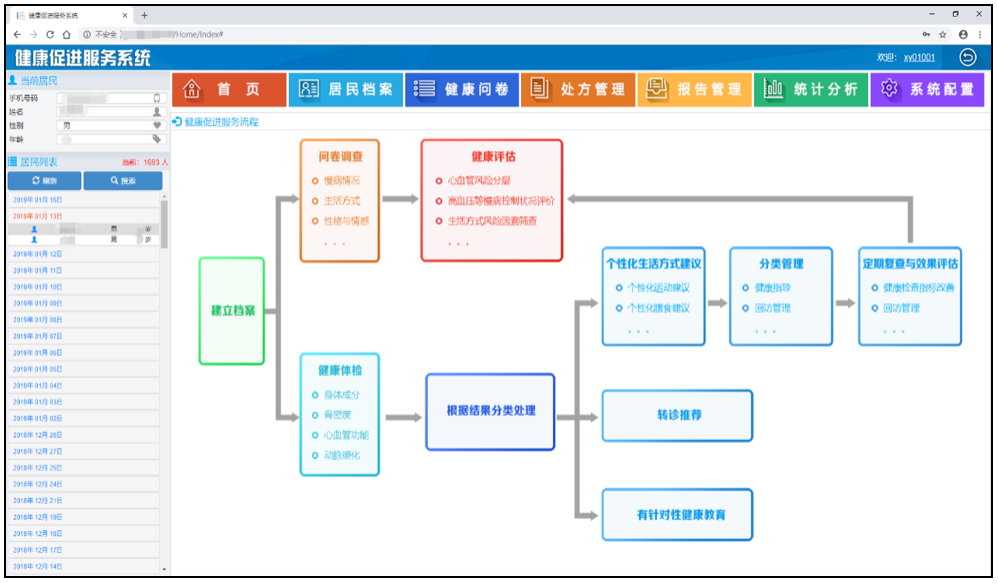
Screenshot of the system’s home page.

**Figure 3:**
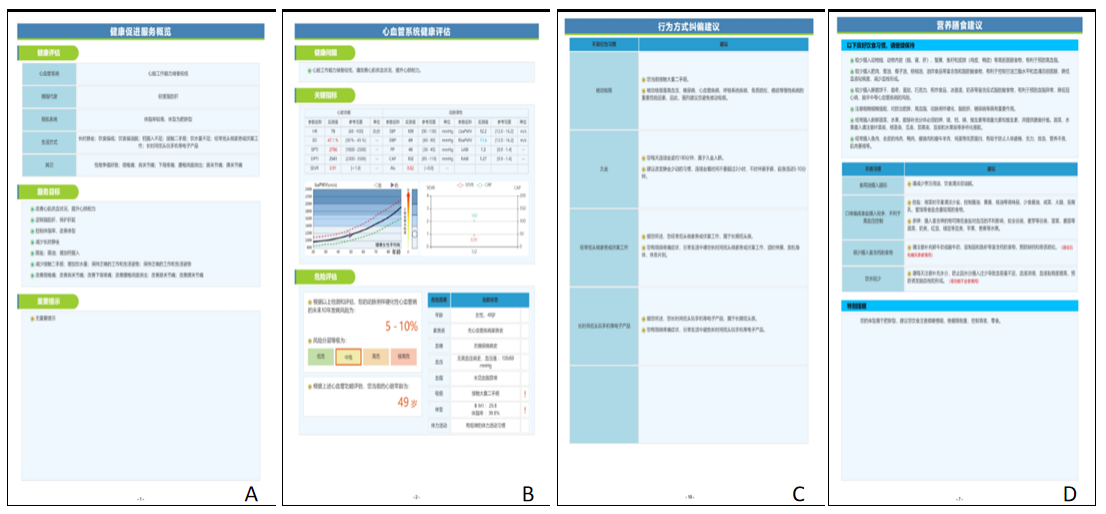
Screenshot of the synthesis report.

The community-oriented intelligent health promotion system, illustrated in Figure 1 for its architecture, the log-in home page shown in Figure 2, and an example of a synthesis report shown in Figure 3, can be conceived as an expert system that offers valuable references for student health education. This system possesses the ability to intelligently produce comprehensive assessment reports by using data from individual health indicators and responses to health questionnaires. In addition, it has the capacity to formulate personalized health guidance plans, inclusive of customized exercise prescriptions.

We have implemented the system in the intelligent health cabin at the community health service centers to generate intelligent and personalized recommendations, which are then used for remote health guidance on WeChat public accounts or other social media platforms. The system currently encompasses four essential components: (1) a registration system, through which participants are typically invited to enroll in the program via a family-contracted physician; (2) a cloud platform that stores and processes all data, enabling remote web-based monitoring of individual data by community health care staff and researchers; (3) internet-based instruments including devices such as cardiovascular function monitors, arteriosclerosis detectors, body composition monitors, bone densitometers, and physical fitness detectors—these instruments are used to assess participants’ cardiovascular function, body composition, bone mineral density, and physical fitness before and after interventions; and (4) an internet-based questionnaire, designed to gather data about physical activity, health-related lifestyle, chronic disease history, medication use, and family disease history. Further information can be found in previously published literature [19].

#### Introduction to SLP-COIHPS

Following the guiding principles of SL organizations such as the National SL Cooperative and the National Youth Leadership Council, we developed SLP-COIHPS:

1. The established service objective entailed delivering noninvasive health assessments to community residents with chronic ailments. Moreover, graduate students used the expert system for health education provision. The educational pursuits encompassed mastery of health assessment techniques, health education skills, improved interpersonal communication, teamwork, and identification of research inquiries within the scope of intelligent health. These inquiries were tackled during service provision, fostering the augmentation, optimization, and expansion of the system’s functionality.
2. During the construction of the intelligent health promotion system and the stages of selecting, designing, implementing, and evaluating service plans, the students’ perspectives were thoroughly considered.
3. For fostering interaction between the academic institution and the community, a trilateral cooperation agreement was formed involving the intelligent health research institute, the university, and the community health service center. Consistent project progress meetings were convened.
4. Before delivering the health services, students underwent standardized training in equipment operation, functioning of the intelligent health promotion system, questionnaire compilation, interpretation of examination outcomes, and health education guided by the system.
5. Scheduled group meetings gathered the medical school faculty, community mentoring instructors, and nursing graduate students. These sessions were anchored in student case studies, facilitating discourse about resolutions to health service–related professional matters. Experiences and insights from professional interactions and engagement with community residents were also shared. Involvement of nursing graduate students commences from the latter half of their first year. After training, they engaged in shifts spanning Monday to Friday, rendering health assessments, educational services, and related duties. This hands-on experience enabled students to discern research inquiries. Upon mentor approval, these inquiries were woven into their master’s thesis research. After verification, they were incorporated into the system.

### Participants

#### Quantitative Study

Until the time of the survey, this study (2018-2022) involved a total of 28 graduate students who enrolled in this university (Bengbu Medical College) from 2017 to 2022. The inclusion criteria for the experimental group in this survey were as follows: (1) they graduated no more than 2 years ago (enrolled in 2019-2022); (2) they had accumulated >6 months of service in the smart houses of community health service centers; and (3) they were willing to participate in the questionnaire survey. The control group was selected using a risk-set sampling method. Individuals in the control group were selected to match each individual in the experimental group in terms of their year of enrollment, sex, and research methodology and having no previous exposure to similar interventions in their graduate studies.

#### Qualitative Study

We used a convenience sampling method to select graduate students from the experimental group who participated in the questionnaire survey and community residents served by these students during the project. Eligibility criteria included the following: (1) community residents who were participants during the project, (2) aged >45 years, (3) clinical diagnosis of at least 1 chronic condition, (4) willingness to participate in qualitative interviews, and (5) adequate language proficiency. The sample size was determined using theoretical saturation. We conducted interviews until thematic saturation was reached, and the last few interviews did not reveal any new patterns or themes.

### Data Collection

#### Quantitative Study

Data were collected between April 28, 2023, and July 30, 2023. Each nursing graduate student received individual Question Star links from a researcher (XX) and, after providing electronic informed consent, responded to the questionnaire. A total of 46 questionnaires were distributed (all 23 graduate students who were currently enrolled in the project or have graduated within the past 2 years, along with their matched 23 counterparts, had provided their consent for the survey), all of which were collected, resulting in a 100% (46/46) response rate. The average completion time was 422.86 (SD 45.31) seconds.

#### Qualitative Study

A researcher (XX) who had received training in qualitative interviewing and was unfamiliar with the interviewees conducted telephone interviews with graduate students who participated in the project. Following the data saturation principle, a total of 15 nursing graduate students ultimately participated in the phone interviews. Postgraduate students were asked, “How has your participation in this program influenced you? What impact has this program had on your key abilities?”

Community residents participating in the interviews were recruited through telephone calls by staff from the community health service center, based on the eligibility criteria. A teacher (TS) trained in qualitative interviewing conducted face-to-face interviews with patients with chronic diseases who had received services from graduate students. The interviews were conducted in the health education room at the community health service center, and no one else was present. Written consent was obtained from the participants, and the interviews were recorded. Interviews continued until thematic saturation was achieved. In total, 12 community residents who had received community health services participated in this study. For community residents, the questions were, “Are you content with this program? Which aspects are particularly satisfactory to you? What changes have resulted from this program for you?”

Before the formal interviews, 2 interviewers (XX and TS) conducted preinterviews with 2 to 3 graduate students and 2 to 3 community residents, respectively. Ultimately, the individual interviews varied in duration from 10 to 30 minutes, with an average duration of 27 minutes. During the interview, the interviewer summarized the answers to the questions, asking whether the summary was accurate and whether anything had been missed. After each interview, the conducting researcher reviewed and listened to the recording multiple times to ensure accurate transcription, and meaning verification was conducted through thorough reading.

### Data Analysis

#### Quantitative Analysis

The quantitative data were analyzed using SPSS statistical software (version 23.0; IBM Corp). General characteristics of the participants were presented as frequencies and percentages and means and SDs. The scores for scientific awareness and research innovation capabilities were also reported as means and SDs. Intergroup comparisons were performed using independent sample *t* tests (2-tailed), and the effect sizes were provided. The Mann-Whitney *U* test was used to compare 2 sets of variables that did not conform to normal distribution. Multivariate analysis of covariance was conducted to determine the effectiveness of the constituent factors of dependent variables on scientific awareness and research innovation capabilities in more depth after controlling for sex, age, and grade. Statistical significance was set at *P*<.05.

#### Qualitative Analysis

We used a constructivist grounded theory approach to analyze the data. The first author (XX) transcribed the interviews verbatim, sought feedback or corrections from participants after the transcriptions were converted to text, and used NVivo (version 12; Lumivero) for data analysis. Then, 2 research team members (TS and XX), who are skilled in qualitative research methods, independently coded each transcript. In the initial stage, the text was reviewed on a line-by-line basis, and segments were coded to categorize comments and passages. Subsequently, these segments were organized into conceptual categories, thereby creating an initial codebook. Then, the researchers applied the initial codebook to the transcripts, iteratively refining and finalizing the categories. Discrepancies were resolved through consensus. If consensus could not be reached, a third researcher (HX) independently coded and made a final decision after comparison and consensus.

### Ethical Considerations

The study was conducted following ethical guidelines and received approval from the ethics committee of Bengbu Medical College (2022-103). Every participant provided written or electronic informed consent and received comprehensive information about the study’s objectives, procedures, interview audio recording, data anonymity, and freedom to withdraw at any point. Personal data and information were treated with strict confidentiality. The participants in this project did not receive any form of material compensation.

## Results

### Participant Characteristics

The mean age of participants in the quantitative study (including the control group) was 24.39 (SD 1.26) years. Among the 46 participants, 42 (91%) were women and 4 (9%) were men. The 46 participants from the 2019 to 2022 cohorts were distributed as follows: 8 (17%), 12 (26%), 14 (30%), and 12 (26%) individuals. Grade, sex, and research methods were used for matching, ensuring an equitable distribution between the intervention and control groups. A total of 15 students participated in the qualitative study, of which 14 (93%) were women. The 15 participants from the 2019 to 2022 cohorts were represented by 3 (20%), 4 (27%), 4 (27%), and 4 (27%) individuals in the respective years. The 12 residents participating in the qualitative study had an average age of 68.83 (SD 5.61; range 57-79) years. Among the 12 residents, 8 (67%) were women. Moreover, of the 12 residents, 10 (83%) individuals possessed a secondary education level, whereas 1 (8%) individual each had primary school and vocational school education.

### Scientific Awareness

The total score for research awareness (*P*<.001) of the postgraduate nursing students participating in the intervention group and their scores in finding problem awareness (*P*<.001), demonstrating the value of problem awareness (*P*<.001), proposing problem awareness (*P*=.002), exploring problem awareness (*P*<.001), and innovating awareness (*P*=.002) were all significantly higher than those of the control group’s postgraduate students. The Cohen *d* and *r* values reflecting effect sizes were greater than 0.8 and 0.37, respectively, for all variables except doubting awareness, indicating a substantial effect size (Table 1).

**Table 1:** Comparison of scientific awareness between the 2 groups.

	Finding problem awareness	Demonstrating the value of problem awareness	Proposing problem awareness	Doubting awareness	Exploring problem awareness	Innovating awareness	Total score for scientific awareness
Experimental group score, mean (SD)	11 (1.83)	10.96 (2.14)	11.78 (2.13)	9.7 (1.99)	10.39 (1.85)	9.78 (2.02)	63.61 (9.3)
Control group score, mean (SD)	8.61 (1.67)	8.3 (1.69)	9.65 (2.27)	8.48 (2.01)	7.96 (1.43)	7.78 (1.74)	50.78 (7.13)
*t* test^a^ (*df*)	4.6 (44)	4.66 (44)	3.28 (44)	2.01 (44)	4.99 (44)	3.6 (44)	5.25 (44)
*P* value	<.001	<.001	.002	.51	<.001	.002	<.001
Cohen *d*	1.36	1.38	0.97	0.61	1.47	1.06	1.55
*r*	0.56	0.57	0.44	0.29	0.59	0.47	0.61

^a^2-tailed t test.

### Research Innovation Capabilities

As the scores of thinking innovation and achievements of scientific research innovation and the total score of research innovation capabilities do not follow a normal distribution, we conducted a Mann-Whitney *U* test, the results of which are presented in Table 2. The overall research innovation capability score of the experimental group was significantly higher than that of the control group (*P*=.004). In addition, scores for thinking innovation (*P*=.004), method innovation (*P*=.04), application innovation (*P*=.001), and achievements of scientific research innovation (*P*=.04) were all high in the experimental group compared with the control group. The values of Cohen *d* and *r* indicate a significant intervention effect of this educational model on thinking innovation, application innovation, and research innovation capabilities (Cohen *d*>0.8; *r*>0.37).

**Table 2:** Comparison of the research innovation capabilities between the 2 groups.

	Thinking innovation	Method innovation	Application innovation	Achievements of scientific research innovation	Total score for research innovation capabilities
Experimental group score, mean (SD)	17.35 (SD 2.93)	3.44 (SD 0.73)	18 (SD 3.06)	6.59 (SD 3.63)	47.17 (SD 17.81)
Control group score, mean (SD)	15 (SD 1.86)	2.96 (SD 0.77)	14.96 (SD 2.42)	3.92 (SD 1.31)	36.61 (SD 10.44)
*t/Z*	−2.87	2.17	3.74	−2.08	−2.89
*P* value	.004	.04	.001	.04	.004
Cohen *d*	0.92	0.51	1.1	0.58	0.94
*r*	0.42	0.25	0.48	0.28	0.43

### Multivariate Analysis of Covariance Testing

The test results of the multivariate Pillai trace in Table 3 show that covariates such as sex (*P*=.62), age (*P*=.8), and grade (*P*=.5) had no statistically significant effect on the dependent variables, namely scientific awareness and research innovation capabilities. The variations in these 5 variables, namely, finding problem awareness, demonstrating the value of problem awareness, exploring problem awareness, application innovation, and achievements in scientific research innovation, were the factors contributing to the differences in the dependent variables. The independent variables collectively account for 93.8% of the variance in scientific awareness and 73.4% of the variance in research innovation capabilities.

**Table 3:** Multivariate analysis of covariance testing.

Effect	Value	*F* test	Hypothesis *df*	Error *df*	*P* value
Intercept	0.36	6.29	2	22	.007
Sex	0.43	0.49	2	22	.62
Age	0.2	0.23	2	22	.8
Grade	0.06	0.72	2	22	.5
Finding problem awareness	0.42	3.09	4	46	.03
Demonstrating the value of problem awareness	0.37	2.65	4	46	*.*045
Proposing problem awareness	0.35	2.42	4	46	.06
Doubting awareness	0.07	0.4	4	46	.81
Exploring problem awareness	0.54	4.26	4	46	*.*005
Innovating awareness	0.33	2.3	4	46	.07
Thinking innovation	0.17	1.03	4	46	.40
Method innovation	0.19	1.15	4	46	.34
Application innovation	0.48	3.68	4	46	.01
Achievements of scientific research innovation	0.58	14.97	4	46	<.001

### Qualitative Analysis of Interviews With Postgraduate Nursing Students

Qualitative analysis revealed 12 subcategories in the following categories: specialized skill, scientific research ability, and comprehensive qualities (Multimedia Appendix 1).

### Qualitative Analysis of Interviews With Community Residents

Qualitative analysis revealed 7 subcategories in the following categories: satisfaction and perceived benefit (Table 4).

**Table 4:** Qualitative analysis of the experience of patients with chronic diseases in the program.

Categories and subcategories		Example statements
**Satisfaction**		
	Credibility of the decision module	“Your personalized exercise plan is undoubtedly well-structured and based on scientific principles. It undoubtedly brings benefits through targeted workouts for specific body areas or overall physical fitness.” [Participant 5]
	Service attitude	“It’s so thoughtful and meticulous of these students to explain the test results to us and guide us on diet and exercise. Isn't it wonderful?” [Participant 11] “Your staff is very meticulous in conducting each of my examinations.” [Participant 6] “...These girls are quite patient. They explain things carefully when I don’t understand. As we’re older and not educated, our speech is unclear, and our hearing isn’t good. You have to repeat each sentence three or four times for us...” [Participant 12]
	Understandability	“...We can understand what they say...” [Participant 7] “...The explanation was very clear...” [Participant 1]
	Professionalism	“They have a wealth of knowledge and communicate fairly well...” [Participant 4] “This report is very clear and comprehensive.” [Participant 9]
**Perceived benefit**		
	Symptom control	“I used to have high blood sugar, but I'm fine now.” [Participant 8] “I used to have constipation, and I still experience it now, though less frequently...” [Participant 2] “I have observed improvements in my health. I originally had high and unstable blood pressure, but now it’s quite stable.” [Participant 3]
	Emotional improvement	“After exercising, I feel a sense of mental ease, and my physical condition has improved somewhat. I also have more energy than before.” [Participant 10]
	Knowledge acquisition	“Compared to before, I now understand the appropriate exercise postures, how to relax after exercising, and how to stretch properly.” [Participant 4] “They informed me that my bone density had reached the baseline and should not decrease further. They instructed us to consume milk, eggs, and certain vegetables daily, and their explanations were thorough.” [Participant 1]

## Discussion

### Principal Findings

This study used a mixed methods approach to investigate the impact of this training model on the research and other skills of nursing graduate students. It also explored the project’s effects from the perspectives of both nursing graduate students and the recipients of their services. Quantitative study findings indicated that this project, incorporating the essential concepts of “medical science and engineering integration,” “intelligent health,” and “service learning,” had a positive impact on enhancing the research awareness and research innovation capabilities of nursing graduate students. Qualitative study findings also demonstrated the favorable influence of the project on improving the scientific research skills of graduate students. This encompassed enhancing their understanding of scientific logic, finding and solving problems, and expanding their critical thinking skills. Moreover, we identified pivotal elements that facilitated the improvement of specialized skills among program participants, including linking reality, web-based coaching, avoiding mistakes, and expanding knowledge. The intelligent promotion system integrated within this project, along with the health services offered by graduate students under its guidance, contributed collaboratively to the perceived benefits experienced by patients. Furthermore, the enhancement of comprehensive qualities among graduate students could potentially exert additional influence on patient satisfaction.

On the basis of our study, findings demonstrated that nursing graduate students who participated in the project exhibited significantly high research awareness and research innovation capabilities compared with their paired control counterparts. With the exception of doubting awareness, these students excelled in aspects such as finding problem awareness, proposing problem awareness, demonstrating the value of problem awareness, exploring problem awareness, innovating awareness, thinking innovation, method innovation, and application innovation. These capabilities were also associated with great achievements in scientific research innovation. The outcomes aligned with similar findings: nursing graduate students with a medical-engineering interdisciplinary background outperformed their counterparts from other disciplines in dimensions such as finding problem awareness, demonstrating the value of problem awareness, and exploring problem awareness (with the control group scoring low) [24]. Furthermore, these students exhibited high scores in terms of overall scientific awareness and application innovation compared with the control group’s nursing graduate students [37].

In this study, except for the aspect of doubting awareness, nursing graduate students who participated in the project demonstrated superior performance in various dimensions of scientific awareness. This distinction might have arisen from the practical teaching model’s ability to inspire students with ideas from diverse disciplines, fostering innovative thinking [38,39]. Qualitative study supplemented this notion: nursing graduate students found it easy to identify and solve problems and enhance scientific awareness through the expert system integrated into this project, along with the provision of health education. However, regarding “doubting awareness,” no significant difference emerged between the 2 groups of students; both tended to seek validation from teachers and experts, rarely challenging their viewpoints. This inclination could be attributed to the traditional Chinese educational emphasis on respecting and learning from teachers, which was hard to change in a short period. Regarding research methodology, the conventional model failed to address the challenges encountered when selecting research topics. Thus, interdisciplinary thinking and methodologies were borrowed to cater to nursing. The cumulative effect of various factors ultimately translated into improved quality and quantity of research outcomes. Therefore, participants in this project attained high scores in dimensions such as “thinking innovation,” “method innovation,” “application innovation,” and “achievements of scientific research innovation” compared with the control group.

SL, an innovative pedagogical approach integrated with community-based intelligent health, plays a significant role in connecting theory and practice, as shown in previous studies [40,41]. By directly interacting with the community, students applied their expertise to solve real-world problems, which was substantially different from the classroom setting. Moreover, SL provided students with the necessary guidance and prompts through a web-based coach based on a community-based intelligent health promotion system, enabling them to accumulate experiential knowledge and avoid potential mistakes. This finding is consistent with those of previous studies [42,43]. Consequently, linking to reality, web-based coaching, mistake avoidance, and knowledge expansion were the key elements of SL based on intelligent health. The “four-in-one” process of practice, reflection, cooperation, and guidance was well integrated with SL, significantly enhancing students’ professional practice skills.

This study revealed that enhancing the general qualities of postgraduate students can have a significant impact on patient satisfaction. Students with strong comprehensive qualities, including effective communication skills, empathy, self-confidence, and professional competence, were more likely to deliver high-quality services and demonstrate professionalism. Their overall excellence directly influenced service quality, professionalism, interpersonal communication skills, and adherence to professional ethics, ultimately leading to positive patient satisfaction. Similar study findings highlighted the positive effects of postgraduate nursing education on students’ knowledge and skills, clinical practice, patient satisfaction, and health outcomes [44]. Furthermore, the study by He [45] demonstrated that improving nurses’ overall quality contributes to enhanced patient satisfaction, reduced incidence of patient complaints, and few nurse-patient disputes. This experience serves as a reference for universities conducting web-based and offline practical teaching in digital health services. Students’ engagement in the entire process of researching, applying, and improving digital products is also a comprehensive practice based on the concept of SL. In addition, the project exposes students to different health service scenarios, addressing the urgent need for references in the field of digital health, such as intelligent health and telehealth education and experience, especially in the current era of increasing technology adoption [27]. This can better assist graduate students in developing the professional skills and overall competence needed in using new technologies.

The findings of this study also support the further research of a practical program that integrates intelligent health and SL theories in the medical education field. This includes investigating potential factors affecting the research abilities of postgraduate nursing students or examining the long-term impacts of research projects.

### Limitations

Limitations emerge owing to the lack of a feasibility study to compare the preintervention and postintervention periods. The study should be conducted on a large sample to authenticate the program’s efficacy while contemplating the process of changes among postgraduate nursing students that are influenced by this program. Future studies should explore diverse samples, considering variations in sex and academic backgrounds. The principles of a randomized controlled trial should be implemented by assigning the study participants to control and experimental groups. Furthermore, it is advisable to conduct longitudinal studies for a thorough assessment of long-term impacts. In addition, a comprehensive evaluation of the impact of community, technological aspects, and cultural influences on the effectiveness of SL programs is recommended.

### Conclusions

SLP-COIHPS had a positive impact on the development of students’ scientific awareness and research innovation ability. Findings of the qualitative study also support the further development of a practical program that integrates intelligent health and SL theories in the medical education field for postgraduate nursing students.

## Appendix

Multimedia Appendix 1 Content analysis of postgraduate nursing students’ experience in the program.

## Abbreviations

SL: service learning
SLP-COIHPS: service-learning project based on a community-oriented intelligent health promotion system

## Data Availability

The data sets generated and analyzed during this study are available from the corresponding author upon reasonable request.
