# Supplementary material for: A Service-Learning Project Based on a Community-Oriented Intelligent Health Promotion System for Postgraduate Nursing Students: Mixed Methods Study
Source: JMIR Med Educ. 2023 Dec 15;9:e52279. doi: 10.2196/52279 (PMC10757228; doi:10.2196/52279)
Supplement: Multimedia Appendix 1 [file mededu_v9i1e52279_app1.doc]

Multimedia Appendix 1. Qualitative analysis of postgraduate nursing students’ experience in the program.

| Categories and subcategories | | Example statements |
| --- | --- | --- |
| **Specialized skill** | | |
|  | Linking reality | - “Although I had studied courses in ‘Community Nursing’ and ‘Health Education,’ I primarily gained theoretical knowledge from textbooks. This was my first experience engaging with patients in the community.” [Participant 11] - “...I realized the concerns of these two groups between community-based chronic disease patients and hospitalized clinical patients are quite different.” [Participant 6] - “I believe that the context of the community is quite distinct from that of the hospital, and there are also certain differences in the required knowledge and skills.” [Participant 5] |
|  | Virtual coach | - “Initially, I felt very nervous. I didn’t know how to conduct health education with patients...I realized that the Intelligent Health System is essentially an expert system that provides me with numerous prompts...for a specific diabetes patient, the system suggested what type of exercises they should do, how many times per week, and the intensity for each session. My role was to explain the reasons behind these recommendations.” [Participant 4] - “Before conducting health services, we underwent training and assessments for the procedures. However, when I started to apply them practically, I still felt nervous. Nevertheless, I noticed that there were clues for each initial step, and the test results also had rough explanations. Even if I encountered individuals with a lower level of education, I could provide explanations based on the prompts.” [Participant 2] |
|  | Avoiding mistakes | - “Many of the community residents who come to us are elderly individuals with complex health conditions. Usually, they don’t have just a single chronic illness. If I were to provide health guidance directly, it would likely be quite challenging. For instance, some individuals have both hypertension and diabetes. Teaching only a diet suitable for hypertension wouldn’t be appropriate...Now, we can utilize AI technology to avoid such mistakes.” [Participant 1] - “I used to think that providing exercise guidance was quite straightforward. However, I’ve come to realize that without this Intelligent Health System, it’s actually quite difficult. Some elderly individuals, due to specific conditions, can’t engage in certain exercises. The intelligent system takes all possible scenarios into account and prescribes exercise routines based on the patient’s habits. This way, I have fewer concerns when offering exercise guidance.” [Participant 11] |
|  | Expanding knowledge | - “The personalized health guidance provided by the Intelligent Health System already takes into account various aspects such as exercise and diet. However, in the process of delivering actual services, these considerations fall far short. You never know what kind of questions community patients with chronic illnesses might have for you. This requires us to continually expand our knowledge base...” [Participant 9] - “They say that experience teaches you, even in the medical field. There were times when patients posed difficult questions, and I couldn’t provide a clear answer. In such situations, I would honestly inform them that I would consult more experienced colleagues and then call them back...” [Participant 8] |
| **Scientific research ability** | | |
|  | Logic of science | - “If we were to merely mechanically follow the prompts provided by the system for health services, it would diminish the significance for us. Understanding the logic behind the system allows me to engage in more dialectical thinking about the issues.” [Participant 3] |
|  | Problem finding | - “Following the previous procedure, after generating reports, we used to provide printed copies to residents. However, elderly individuals tend to forget, and they often can’t find the reports once they return home. This got me thinking, could we create a mobile platform so they can view the reports anytime? Now, I need to contemplate how to initiate a mobile health project.” [Participant 7] - “The exercise prescription provided by this system is already quite clear, but why do chronic disease patients still exhibit poor adherence? Which is more effective, personalized exercise guidance or group exercise guidance? I intend to design a study to verify this.” [Participant 12] |
|  | Problem-solving | - “I observed that the dietary guidance provided by the system to diabetic patients is too general, and some patients cannot fully comprehend it. This led me to consider whether there might be a more effective approach to health education. As a result, I conducted an extensive literature review, identified a theoretical framework, and devised an intervention process. The current results suggest a favorable outcome.” [Participant 10] |
|  | Broadening of thinking | - “As a medical student, I used to perceive AI as distant and had never considered its potential applications for me. Now that I comprehend our system, I realize that I can view it as a tool. While AI aids intelligent decision-making, the essence of sound logic still rests with medical students. You know, this cannot be entirely entrusted to computers...” [Participant 15] - “I might be more intrigued by the instrument devices interconnected within the system, or to be more precise, I am interested in identifying a more affordable, non-invasive medical indicator to substitute for invasive gold standards. This approach could facilitate the development of a universally applicable screening tool.” [Participant 13] |
| **Comprehensive qualities** | | |
|  | Sympathy | - “She shared with me the suffering that chronic illness has brought to her body. What surprised me was that she also carries immense emotional stress. She described herself as feeling like rubbish, while other elderly individuals can engage in activities that contribute to their families or enjoy outdoor excursions, and yet she feels incapable of doing anything. This deeply moved me, providing my first profound insight into how disease impacts more than just the physical realm.” [Participant 5] - “I’ve observed that some elderly individuals experience considerable loneliness in their later years. They truly require the care and attention of people from all walks of society...” [Participant 14] |
|  | Communication ability | - “One of the residents scheduled for a health examination didn’t arrive at the appointed time. While I was providing service to others, he displayed impatience. In the past, encountering such individuals would frustrate me. Now, I can calmly and patiently explain our procedures and reassure him to wait in line...” [Participant 12] - “I used to struggle with social anxiety and had difficulty conversing with others. Now, I’ve developed some strategies. For instance, if some ladies have a great physique, I compliment them before conducting examinations and ask about their usual physical activities. This is also integrated into the questionnaire.” [Participant 3] |
|  | Professional role | - “Since we conduct follow-up visits with residents every three months, many patients have become familiar faces. They place significant trust in me and willingly heed the advice I provide, which gives me a strong sense of satisfaction.” [Participant 7] - “A resident shared with me that after following my dietary and exercise recommendations, her blood pressure has remained remarkably stable. I am truly delighted, as my expertise has positively impacted someone's health.” [Participant 6] |
|  | Self-confidence | - “Before the project commenced, I used to worry about making mistakes. However, with the increasing number of health service sessions, I gradually transitioned from relying entirely on the expert system. Now, I have gained more and more knowledge, and I find myself increasingly adept.” [Participant 10] - “I encountered some patients who raised questions beyond the scope of expert system knowledge. To address these, I conducted research and sought advice from instructors, ultimately providing them with satisfactory answers. This process bolstered my confidence.” [Participant 6] |
